# Continuous monitoring of surgical bimanual expertise using deep neural networks in virtual reality simulation

**DOI:** 10.1038/s41746-022-00596-8

**Published:** 2022-04-26

**Authors:** Recai Yilmaz, Alexander Winkler-Schwartz, Nykan Mirchi, Aiden Reich, Sommer Christie, Dan Huy Tran, Nicole Ledwos, Ali M. Fazlollahi, Carlo Santaguida, Abdulrahman J. Sabbagh, Khalid Bajunaid, Rolando Del Maestro

**Affiliations:** 1 Neurosurgical Simulation and Artificial Intelligence Learning Centre, Department of Neurology & Neurosurgery, Montreal Neurological Institute, McGill University, 3801 University Street, Room E2.89, H3A 2B4 Montreal, Quebec Canada; 2 Department of Neurology and Neurosurgery, Montreal Neurological Institute and hospital, McGill University, Montreal, Quebec Canada; 3 Division of Neurosurgery, Department of Surgery, College of Medicine, King Abdulaziz University, Jeddah, Saudi Arabia; 4 Clinical Skills and Simulation Center, King Abdulaziz University, Jeddah, Saudi Arabia; 5 Department of Surgery, Faculty of Medicine, University of Jeddah, Jeddah, Saudi Arabia

**Keywords:** Health care, Medical research

## Abstract

In procedural-based medicine, the technical ability can be a critical determinant of patient outcomes. Psychomotor performance occurs in real-time, hence a continuous assessment is necessary to provide action-oriented feedback and error avoidance guidance. We outline a deep learning application, the Intelligent Continuous Expertise Monitoring System (ICEMS), to assess surgical bimanual performance at 0.2-s intervals. A long-short term memory network was built using neurosurgeon and student performance in 156 virtually simulated tumor resection tasks. Algorithm predictive ability was tested separately on 144 procedures by scoring the performance of neurosurgical trainees who are at different training stages. The ICEMS successfully differentiated between neurosurgeons, senior trainees, junior trainees, and students. Trainee average performance score correlated with the year of training in neurosurgery. Furthermore, coaching and risk assessment for critical metrics were demonstrated. This work presents a comprehensive technical skill monitoring system with predictive validation throughout surgical residency training, with the ability to detect errors.

## Introduction

The mastery of technical skills is of fundamental importance in medicine and surgery as technical errors can result in poor patient outcomes^1–3^. The learning of bimanual psychomotor skills still largely follows an apprenticeship model: one defined by a trainee completing a fixed-length residency working closely with instructors. Technical skills education is transitioning from this time-focused approach to competency-based quantifiable frameworks^4,5^.

Surgical trainees are considered competent when they can perform specific surgical procedures safely and efficiently, encompassing knowledge, judgement, technical and social skills to solve familiar and novel situations to provide adequate patient care^6^. The focus on “adequate” rather than “excellent” or “expert” patient care relates to challenges in outlining, assessing, quantifying, and teaching the composites of surgical expertise. To provide competency-based frameworks for complex psychomotor technical skills, advanced platforms need to be created which provide objective feedback during training along with error mitigation systems^7^. These frameworks need to be transparent and based on quantifiable objective metrics^8,9^.

A technically challenging operative procedure in surgery involves the subpial resection of brain tumors adjacent to critical cortical structures^10^. Neurosurgical graduates are expected to be proficient in this complex bimanual skill which includes minimizing injury to adjacent normal tissues and hemorrhage from subpial vessels. Technical errors in this procedure can result in significant patient morbidity^10,11^. Our group developed complex realistic virtual reality tumor resection tasks to aid learners in the mastery of this skill^12,13^. Exploiting these simulations on the NeuroVR platform with haptic feedback (CAE Healthcare, Montreal, Canada) we quantified multiple components of the bimanual psychomotor skills used to expertly perform these tasks. Utilizing this data post-hoc, we developed expert performance benchmarks to which learner scores were compared and machine learning algorithms to classify participants into pre-defined expertise categories^8,14,15^. Limitations of these applications were the inability of ongoing assessment and error detection and improving performance during the task by providing continuous feedback.

Most surgical skills learning occurs in the operating room, with the surgeon instructor continuously evaluating trainee performance and providing coaching to improve performance with a particular focus on preventing surgical errors which may cause patient injury. This assessment occurs in real-time and is relevant to the precise action being performed by the trainee and the risks associated with that action. To mimic the role of expert operative instructors, we developed an artificial intelligence (AI) deep learning application, the Intelligent Continuous Expertise Monitoring System (ICEMS). The ICEMS was developed with two objectives: 1) to make a continuous assessment of psychomotor skills to detect less-skilled performance during surgery, 2) to provide ongoing action-oriented feedback and risk notifications.

**Fig. 1:**
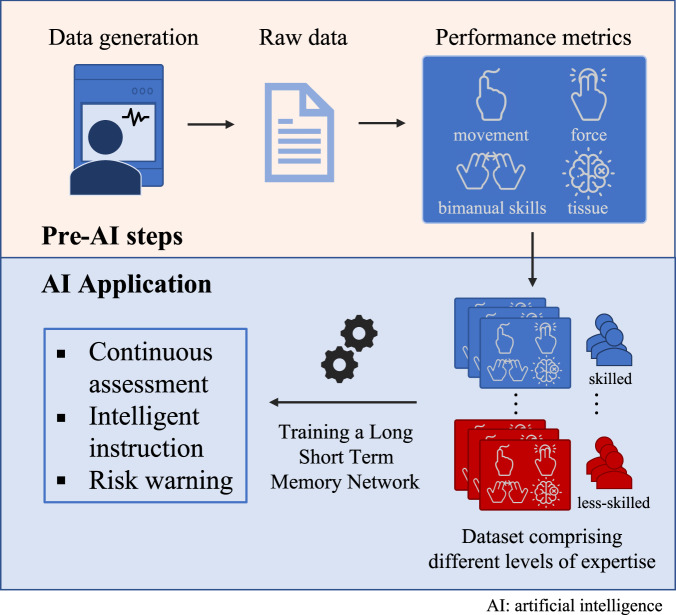
Outline of the application. Raw data acquired from the simulator is used to calculate relevant features, metrics of interest. Data obtained from participants who are at different stages of expertise is used to train a LSTM network. The trained algorithm provided continuous assessment, intelligent instructions, or risk warnings, depending on the output feature selected. Multiple algorithms are trained to demonstrate potential applications of the ICEMS.

This paper outlines the development of the ICEMS (Fig. 1) and provides predictive validation evidence that enables future studies to explore its efficacy in simulation training. To our knowledge, this application is the first continuous bimanual technical skill assessment using deep learning with the predictive validation on surgical trainee performance throughout a residency program^16^.

## Results

### Participants and data

**Fig. 2:**
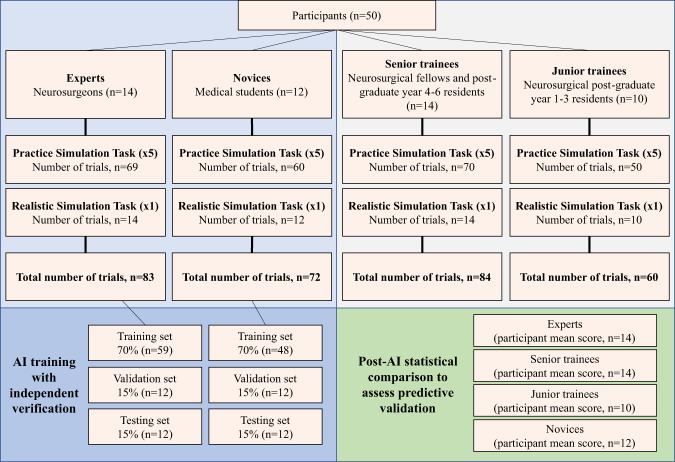
Flow diagram. AI: artificial intelligence. One trial data belonging to a neurosurgeon was not available.

**Fig. 3:**
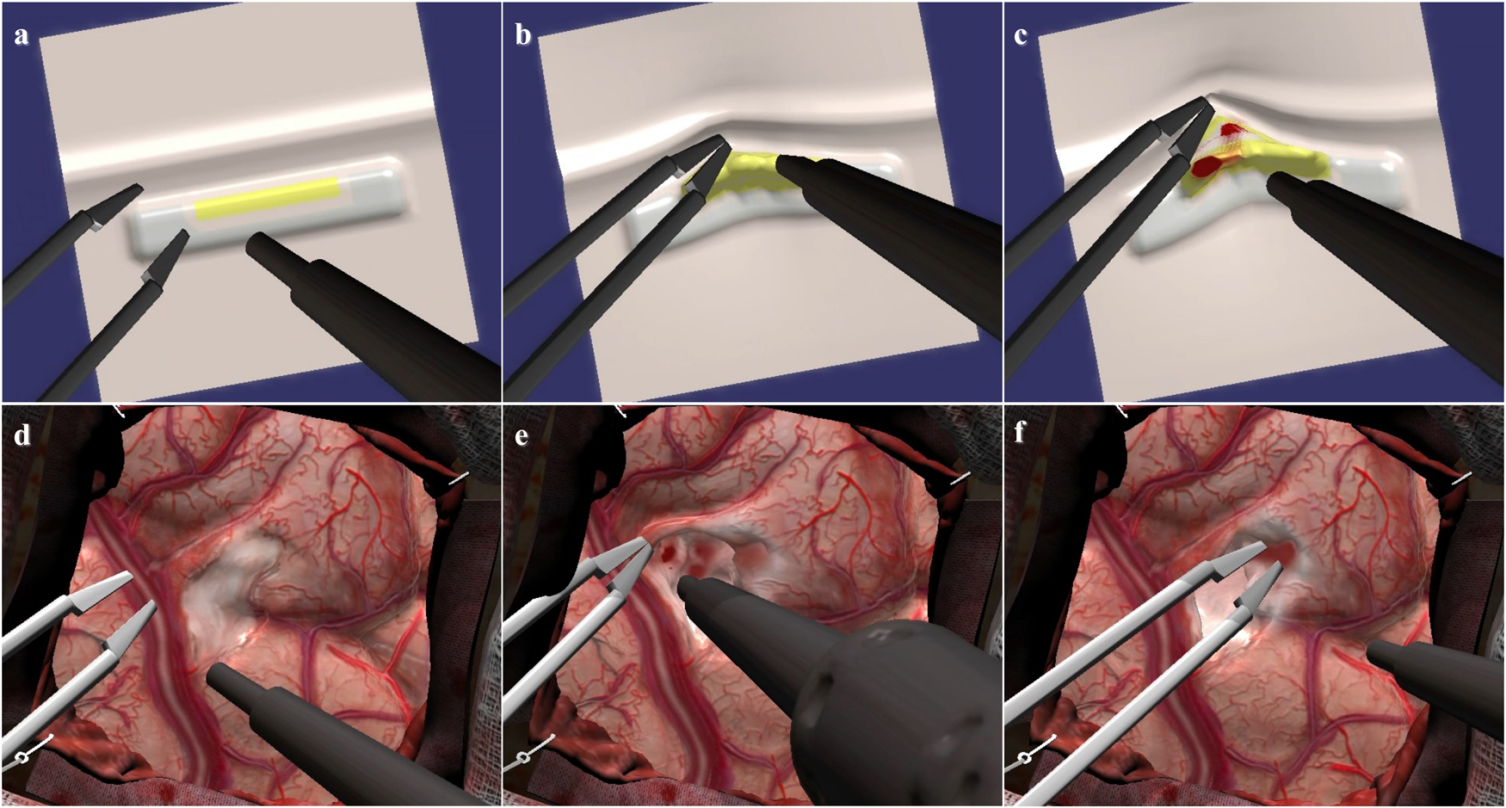
Simulated tumor resection tasks. Participants carried out two simulated tumor resection tasks, the simulated subpial tumor resection (**a**, **b** and **c**) 5 times and the simulated complex brain tumor operation (**d**, **e** and **f**) once, employing a simulated ultrasonic aspirator in the dominant hand and a simulated bipolar forceps in the non-dominant hand. Both instruments were activated by separate pedals. These tasks were designed with bleeding capacity to replicate the high-risk complex subpial brain tumor resection. (**f**) demonstrates cauterization using the bipolar forceps.

Neurosurgeons, neurosurgical fellows, neurosurgical residents, and medical students from McGill University were invited to participate. Neurosurgeons and medical students were categorized as experts (*n* = 14) and novices (*n* = 12), respectively. Neurosurgical fellows and residents were allocated a priori into two groups based on their previous operative exposure: seniors (4 neurosurgical fellows and 10 neurosurgical residents in years 4–6,) and juniors (10 neurosurgical residents in years 1–3) (Table 1). Each participant performed two different simulated subpial tumor resection tasks a total of six times, resulting the data from 300 attempts in total (Fig. 2). The simulated scenarios were described previously (Fig. 3)^8,12^. Data were recorded in a single time point. No data-exclusion was applied. Mean age [SD] was, for experts: 45.9 [8], for seniors: 32.3 [2.1], for juniors: 29.8 [3.2] and for novices: 24 [1.3]. Trainee number of complete subpial tumor resections performed (mean [min-max]) was, seniors: 14.7 [0–45], juniors: 1 [0–7] (Supplementary Table 3).

**Table 1:** Residents’ demographics.

	Post Graduate Year of Training	Number of Trainees
Neurosurgical Fellows	7	4
Neurosurgical Senior Residents	6	3
	5	2
	4	5
Neurosurgical Junior Residents	3	4
	2	2
	1	4
Total		24

Twenty-four neurosurgical trainees participated in the study: 4 neurosurgical fellows, 10 senior residents (post-graduate year 4–6), 10 junior residents (postgraduate year 1–3).

### AI design and development

The definition of expertise in surgical technical skills is challenging since surgical performance involves continuous interplay between multiple factors^17^. However, the composites of expertise are present in the performance of expert professionals. We developed the Intelligent Continuous Expertise Monitoring System in this context by training a Long-Short Term Memory (LSTM) network to learn operative surgical expertise from the difference between expert and novice surgical skills considering the continuous flow of the performance. The algorithm was trained with both end skill levels with more than 700 min of operative performance with a data entry at 0.2-s intervals (with over 200,000 data points of analysis).

A surgical performance is a combination of multiple intraoperative interactions. An appropriate assessment requires considering these tasks being carried out within the flow of the performance. LSTM networks, as a type of recurrent neural network, allowed for the evaluation of each time point in relation with the previous time points, giving the ability to consider sequences in movements^18–20^.

**Fig. 4:**
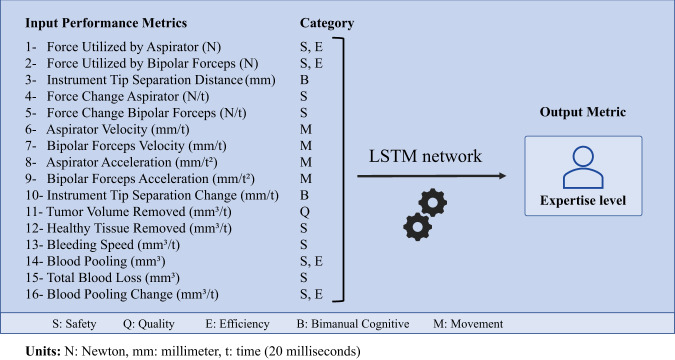
Performance metrics. Sixteen performance metrics from five categories: safety, quality, efficiency, bimanual cognitive and movement, were extracted from the raw data. An LSTM network was trained inputting the 16-performance metrics, predicting expertise. The LSTM network was structured as regression model to predict expertise as a continuous variable from 1 (expert) to −1 (novice). N Newton, mm millimeter, t time (0.02 s).

Sixteen performance metrics were extracted at 0.2-s increments from the simulation data (Fig. 4). Metrics included features related to bimanual technical skills such as instruments tip separation distance, force applied by each instrument and velocity and acceleration of each instrument as well as operative factors such as tumor removed, control of bleeding and damage to healthy tissue. An LSTM algorithm was built by inputting these 16-performance metrics utilizing only expert/neurosurgeon (*n* = 14) and novice/medical student (*n* = 12) performance data on 84 and 72 tasks, respectively. The algorithm was structured as a regression model quantifying expertise level as a continuous variable from expert/skilled level (a score of 1.00) to novice/less-skilled level (a score of −1.00). To avoid overfitting, root-mean-squared-error (RMSE) values on the three separate datasets were monitored (Supplementary Table 1). Detailed information about algorithm structure and development can be found in Online Methods and Supplementary data.

### Quantifying skills

The performance of 24 trainees (on 144 tasks) in different years of neurosurgery training (Table 1) was used to assess the algorithm’s predictive validation. All 300 participant trials were scored by the trained LSTM algorithm at 0.2 s intervals between ‘1.00’(skilled) and ‘−1.00’(less-skilled). An average performance score was calculated for each task (Supplementary Fig. 5). Participants’ mean scores were calculated across six trials for statistical comparisons.

Group average surgical performance scores were; experts, 0.509; 95% CI [0.424–0.593]; seniors, 0.258; 95% CI [0.114–0.402]; juniors, −0.11; 95% CI [−0.358–0.139]; and novices, −0.398; 95% CI [−0.545–−0.251]. No outliers were found, as assessed by boxplot. Only a trial data that belongs to a fifth attempt of a neurosurgeon was missing, no imputation was made. Average performance score was normally distributed for each expertise group as determined by Shapiro-Wilk test (*p* > 0.05). Levene’s test showed equality of variances, based on median (*p* = 0.083).

**Fig. 5:**
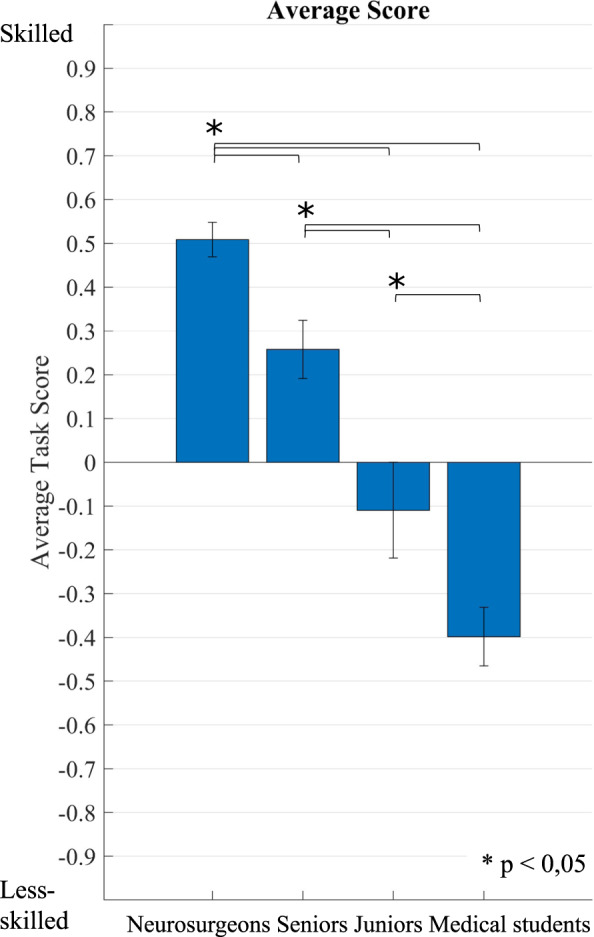
Average score of groups. When the performance of the participants was scored by the ICEMS, the average scores were: for experts (neurosurgeons, *n* = 14) 0.509; 95% CI [0.424–0.593], for seniors (*n* = 14) 0.258; 95% CI [0.114–0.402], for juniors (*n* = 10) −0.11; 95% CI [−0.358–0.139], and for novices (medical students, *n* = 12) −0.398; 95% CI [−0.545 −0.251]. Skilled and less skilled performance are represented in the y-axis by scores closer to ‘1’ and ‘−1’, respectively. Bars represent standard errors.

**Fig. 6:**
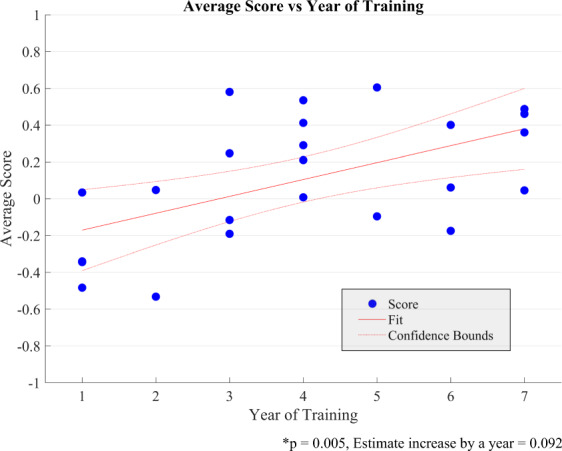
Average score versus year of training in neurosurgery. The average score yielded a significant correlation with the trainees’ year of training (*p* = 0.005), increased by 0.092 per training-year, with a linear regression analysis. Blue dots represent the average score of each trainee, x axis represents year of training in neurosurgery. Resident participants’ neurosurgery training program was six years. Neurosurgical fellows were considered in 7th year in training.

The average performance score was significantly different between expertise groups, *F*(3,46) = 33.927, *p* < 0.001, as determined by a one-way ANOVA. Tukey-Kramer post-hoc test of between groups differences revealed that the expert group scored significantly higher than seniors (mean difference: 0.251 95%CI [0.004–0.497], *p* = .045) and juniors scored significantly higher than novices (mean difference: 0.289 95%CI [0.009–0.568], *p* = .04) in average performance score. The ICEMS also differentiated between surgical trainee groups with seniors scoring significantly higher than juniors (mean difference: 0.367 95%CI [0.097–0.638], *p* = .004) (Fig. 5). In a linear regression analysis resident year of training in neurosurgery statistically predicted the average performance score, *F*(1, 22) = 9.81, *p* = 0.005 and accounted for 30.8% of the variation in the average score with adjusted R2 = 27.7%, a large size effect according to Cohen (1988)^21^. Average performance score increased by 0.092, 95% CI [0.031–0.153] per training year (Fig. 6). The ability of the ICEMS to continuously assess surgical performance during the surgical task is demonstrated in videos outlining a neurosurgeon [video-1] and a medical student performance [video-2] (video legend: Supplementary Fig. 3).

### Coaching and risk detection

A major application of the ICEMS is to provide continuous personalized action-oriented feedback helping trainees modify their bimanual psychomotor movements to expert-level performance and provide critical information to mitigate errors. Three algorithms provided continuous expert-level coaching for (1) aspirator utilization, (2) bipolar forceps utilization and (3) bimanual coordination^8,15,22^. These algorithms provided the ability to revise instrument utilization to expert level continuously. Two other algorithms demonstrated ongoing risk detection capacity for (4) bleeding and (5) healthy tissue injury^8,23^. RMSE values obtained for training, validation and testing of these algorithms are available in Supplementary Table 1.

Although, the validation of these modules in practice for coaching and risk detection will be the object of future studies, we outline the video performance of these algorithms on a senior [video-3] and a junior resident operation [video-4] (video legend: Supplementary Fig. 4). Learning from the difference between expert and novice performance, the ICEMS reproduces some components of intelligent assessment and coaching similarly provided by expert surgical instructors in the operating room.

## Discussion

The transition towards competency-based quantifiable frameworks for evaluation and teaching of surgical technical skills is resulting in the development of high-fidelity virtual reality simulators to aid this learning transformation. These systems provide trainees with repetitive opportunities for experiential learning in patient risk-free environments without limitations imposed by the availability of expert surgical instructors or patient cases^24–26^. We demonstrate an artificial intelligence application to enable these platforms to function as objective autonomous intelligent training platforms with the ability to continuously track psychomotor learning as surgical trainees transition along the spectrum from novice to expert performance.

The NeuroVR platform (previously NeuroTouch, CAE Healthcare, Montreal, Canada) used in this study is a high-fidelity virtual reality neurosurgical simulator that allows 3D visual and haptic interaction in a hyper-realistic simulated surgical environment^13^. This platform was developed by a team of engineers from the National Research Council of Canada with expert inputs from 23 international training hospitals. Extended realism was provided by the 3D microscopic visualization through a binocular, and two haptic handles to allow bimanual simultaneous movement. Tumor physical properties were adjusted using data from multiple primary human brain tumor specimens^27^. Haptic tuning was applied based on the feedback from neurosurgeons^12^. Human brain tissue and bleeding mechanics were implemented including pulsation of blood vessels. A brain tumor surgery intraoperative audio recording was added to increase background auditory realism. The vast dataset generated by this platform allowed for the development of comprehensive intelligent systems^8,9^.

Studies involving real-time surgical technical skills assessment demonstrated supportive results; however, these studies were restricted to one-handed virtual reality systems during a steerable needle task, epidural needle insertion or drilling a simulated femur^28–30^. Most operative procedures involve the coordinated interactions of both hands, each employing a different instrument to accomplish an operative goal. The major roles of expert operative room surgical instructors are to assess trainees’ bimanual skills and help them improve their skills to safely carry out procedures to decrease patient morbidity and mortality^31^. This is crucial especially for high-risk medical procedures. Our group has focused on developing an LSTM network to mirror the role of surgical instructors in assessing bimanual performance involving high-risk complex neurosurgical procedures like the subpial resection. Previous real-time assessment applications utilized small datasets, included engineering students or nonidentified participants and have not validated or tested their algorithms on appropriate learner performance^16,28–30,32^. In contrast, the ICEMS was developed utilizing neurosurgeon/expert and medical student/novice performance, and its performance was tested using the data from neurosurgical trainees who are at different stages of training.

Our framework offers several advantages. First, the ICEMS was trained as a regression model with the two-end skill level performance, providing a continuous expertise scale from novice to expert level. This allowed a more granular performance assessment from the previous applications^8^ and tracking of learning throughout the years of residency training from medical school training to years of practice. Second, we developed our system utilizing two simulated tasks that require the same bimanual surgical technique. This approach offers a more generalizable assessment of this surgical technique across different tasks.

One of the drawbacks of deep learning applications is the ‘black box’ problem where the complexity of the analysis (1) limits the interpretability of the assessment and (2) makes providing relevant information for feedback difficult. To overcome these issues; (1) our assessment system was built on relevant features that are easy to understand and learn. Based on our previous studies, we implemented features representing dominant and non-dominant hand movement and force applied, bimanual cognitive, tissue and bleeding information, and safety metrics. (2) Separate algorithms were trained to work in reverse and provide ongoing feedback for the very features that the assessment was made on. We demonstrate a methodology to generate feedback for any essential performance metric and provide five example features for coaching and risk detection (Supplementary Fig. 2).

In previous self tutoring frameworks, the proposed coaching was based on expert level classification or pre-recorded expert parameters such as videos, benchmarks, or milestones^9,33–35^. In contrast to determining feedback based on expertise group classification or static parameters, the ICEMS produces dynamic feedback for each performance metric by separate algorithms. This involves revising predictions to the highest expert performance level for specific metrics continuously throughout the task, and this revised information can be used as feedback for trainees or any level of performance including expert groups. An action-oriented personalized coaching is provided for specific metrics.

The continuous evaluation done by the ICEMS can be utilized either in real-time to produce visual, auditory, and haptic clues to enhance performance during the task, or to make a summative assessment and provide feedback after task completion. Both learners and instructors can be provided with post-hoc performance videos flagged with the exact time frame(s) of less-skilled performance (see the videos provided in Results). This AI-generated information outlining the reasons for less-skilled assessment may improve trainee self-directed performance and help educators improve learner skills.

Experts may demonstrate performance features that are similar to that of less-skilled level performance. These common features may be due to the intrinsic characteristics of human bimanual performance, the simulated task, or the limits in recording data. For this reason, the ICEMS was built using expert-level performance in comparison to novice performance to differentiate expert specific features. Our results have shown that these expert-specific patterns were increasing throughout trainee-years in training.

Expert surgeons develop and implement autonomous motor activity defined as ‘psycho-motor skills script’ with increasing surgical knowledge^15^. Our system allows trainees to have constant awareness of their level of performance as visualized on a less-skilled to expert scale. By self-modifying their bimanual psychomotor movements with the capacity for unlimited repetitions to achieve expert performance trainees may more quickly develop a “psychomotor skills script” associated with muscle memory that expert surgeons develop and maintain. This may allow trainees to be more prepared when faced with similar procedures in the operating room^15,36,37^.

Our system is developed in the context of surgical simulation using the extensive information recorded by a specific virtual reality simulator. However, this methodology can be useful beyond the scope of surgical simulation and applicable to any technical performance where the necessary data is available. Intraoperative surgical instrument tracking systems are being developed^27^. Future surgical operative rooms may benefit from this application by the integration of AI and intraoperative data recording systems/instruments^38,39^. Surgical operative rooms may evolve into intelligent operating rooms outfitted with a series of evaluating and intelligent tutoring platforms focused on enhancing safe operative performance and thus improving patient outcomes^40^.

Studies have demonstrated that technical skills may correlate with surgical outcomes^2,41^. Improvement in technical skills may improve the outcome, hence, current attempts in simulation training are focused on enhancing trainee technical skills acquisition. However, it remains to be explored if training with intelligent simulation systems can improve patient outcomes.

Deep learning applications require larger datasets^19^. Complex patient cases often require surgeons who have specific expertise in these operative procedures. Surgical trainees acquire these skills operating with limited number of experts, but in multiple repetition of patient cases. Intelligent systems can be developed in a similar way that the trainees learn, using information from limited number of experts but involving multiple occasions of a surgical procedure. This study involved data from 14 neurosurgeons (experts) each repeating the simulation tasks a total of six times, allowing an assessment of 83 expert trial data. If the number of experts is limited, the number of task repetitions performed by each surgeon can be increased to develop accurate and generalizable intelligent systems. This approach may provide a feasible and reproducible method in the intelligent assessment of different surgical skills. Should the data size be limited, data augmentation methodologies can help to increase data size and achieve reliable predictions^42^. Intelligent systems can be continuously improved with more data available. Applications with real-time assessment, coaching and risk detection ability may promote the use of these systems, provide access to new data, and allow further improvement of these systems.

This study has several limitations. Our simulation does not reproduce many of the complex and dynamic learning interactions occurring in modern operating rooms and variables such as the view angle, surgeon instrument choice and instrument intensities were controlled. As simulation platforms advance and incorporate more detailed real-life interactions, more comprehensive assessments can be generated by the ICEMS. For training this supervised deep learning application, each data point of the performance of expert and novices was given the same score (expert: 1.00, novice: −1.00) throughout the task, allowing the algorithms to learn both extremes of the skill spectrum. However, individuals may not always perform in line with their expertise levels. In other words, skilled individuals may perform closer to less-skilled level in certain parts of the procedure and vice versa. Nevertheless, the magnitude of the data allowed algorithms to learn from the two end-skill levels and our system provided a granular differentiation across expertise levels as well as between trainee levels. We defined trainee expertise level based on operative exposure or year in training. However, trainee skill levels may not be completely consistent with these parameters and many other factors may also affect trainee technical skill, including trainee inherent ability or the type of exposure to operative skills^23^ (Supplementary Table 3). By quantifying skills, our application addresses an important limitation for future studies to track trainee learning and explore trainee learning patterns^43^. Our study involved small number of participants from a single institution. With a broader cohort, the generalizability of our model can be increased.

This work, being limited to a previously collected data, provided a validation for the assessment module. An ongoing randomized control trial (ClinicalTrials.gov Identifier: NCT05168150) is addressing the efficiency and validation of coaching and risk detection modules by providing feedback to trainees while tracking their improvement by the assessment module.

As newer technologies^44^ and techniques such as reservoir computing^45,46^ become available, further progress can be made in the applications of continuous technical skill assessment, feedback and operative risk detection using newer and existing datasets.

With the ongoing pandemic, limiting human contact became an essential practice and the present educational paradigms are being re-evaluated^47^. Virtual reality simulators provided with assessment and coaching modules are self-practicing intelligent tools, which may aid trainees and educators navigate the ever-evolving landscape that learners will face.

This work presents a technical skills continuous assessment application built using expert surgeon data, with predictive validity across a training program on surgical trainee performance^16,35^. This deep learning application demonstrated a granular differentiation across expertise and between resident levels. The ICEMS offers a generalizable and objective continuous assessment of surgical bimanual skills which may have implications in the assessment and training of procedural interventions.

## Methods

### Setting

Data of this consecutive retrospective case series study was collected at a single time point between March 2015 to May 2016, with no follow-up. Neurosurgeons, neurosurgical fellows, and residents from one Canadian university were invited to participate in this study at the Neurosurgical Simulation and Artificial Intelligence (AI) Learning Centre, McGill University. Medical students who expressed interest in neurosurgery or were rotating on the neurosurgical service were also invited to take part. Participant data was anonymized. All procedures followed were in accordance with the ethical standards of the responsible committee on human experimentation (institutional and national) and with the Declaration of Helsinki^48^. This study was approved by the McGill University Health Centre Research Ethics Board, Neurosciences-Psychiatry and all participants signed an approved consent form before trial participation. This report adheres to guidelines for best practices in reporting studies on machine learning to assess surgical expertise in virtual reality simulation, reporting observational studies and the reporting of studies developing and validating a prediction model, as applicable^49–52^.

### Simulation

Participants carried out a simulated subpial tumor resection 5 times followed by a simulated complex brain tumor resection (Fig. 3), employing a simulated ultrasonic aspirator in the dominant hand and a simulated bipolar forceps in the non-dominant hand, using the NeuroVR high-fidelity simulation platform (CAE Healthcare, Montreal, Canada). These tasks were designed to replicate the high-risk complex subpial brain tumor resection task^12^. Participants were given verbal and written instructions to remove the tumor completely while minimizing bleeding and injury to surrounding tissue. Simulation data was recorded by the NeuroVR platform in 0.02-s increments (50-recording per second).

### Performance metrics

Before any processing, the raw data underwent interpolation to regularize the timing of data points. Sixteen performance metrics were extracted from raw simulation data, at 0.2 s intervals, based on our previous studies, representing five essential aspect of the operative performance: safety, quality, efficiency, bimanual cognitive and movement^14,23,31,53–59^. Although, deep learning does not require metric extraction, The ICEMS is developed as a training and feedback tool, therefore particular attention is given to develop the system on features which a trainee can understand and learn. The performance metrics are listed in Fig. 4.

### Data preparation before AI application

The data comprised a total of 156 tasks (neurosurgeons: 84 tasks, medical students: 72 tasks) was randomly divided into three different subsets as training (70%, a total of 107 tasks), validation (15%, a total of 24 tasks) and testing (15%, a total of 24 tasks) dataset, to provide independent verification and validation (Fig. 2)^60^. Each individual’s performance data was always kept in the same subset. The performance metrics were normalized by z-score normalization, using the mean and standard deviation values based on the training set. Since the algorithm was designed as a ‘regression’ model where the output feature is predicted as a continuous variable, the categories of expertise levels were transformed into numbers where neurosurgeons (experts) and medical students (novices) were represented as ‘1’ and ‘−1’ respectively, at 0.2-s intervals. Assessment could be as frequent as 0.02 s (50 decisions a second) however we limited the decisions to 0.2 s (5 decisions per second) as more frequent decisions may overwhelm human perception. Considering the z-score normalization, ‘1’ and ‘−1’ represented one standard deviation above and below the mean performance, these values determined the two end of the performance (expert versus novice) of neurosurgical skill. This arrangement allowed not only detecting the two end levels of surgical performance but also the assessment of the performance spectrum in between.

### Algorithm design and AI training

Long-short term memory (LSTM) network is favorable for time-series performance analysis where long-term relations are important^18–20^. We utilized a supervised learning technique and designed our algorithm as a regression model. Our LSTM network was designed to minimize the computational burden (Supplementary Fig. 1). The algorithm composed the first input sequence layer, two unidirectional LSTM layers, a fully connected layer, and a regression layer. Two dropout layers were used, after each LSTM layer, to help avoid overfitting. The number of nodes used for LSTM layers was calculated by adding one (1) to the number of input metrics (performance metrics). Sequence-to-sequence supervised learning was used. More complex designs can be developed, and the performance can be compared to our design. During the training, Adam (adaptive moment estimation) optimizer was utilized with a starting learning rate of 1e-3, decreased by x0.1 every 25 epochs. Minibatch size was 18, determined as the number of trials in the training set (108) divided by the number of repeats per person (6). Shuffling was used at every epoch. The training was performed with 1000 epochs monitoring root-mean-squared-error values visually (Supplementary Table 1), using NVIDIA GeForce GTX 660 (6.0 Gbps).

### Assessing trainee performance

The trained algorithm was used to make an assessment at 0.2-s intervals considering 16 performance metrics. Assessment was made as a continuous variable from ‘1’ expert level to ‘−1’ novice level while any score above ‘1’ or below ‘−1’ was also allowed. The data from 24 neurosurgical trainee participants (six trials per participant) on 144 tasks was used to test the algorithm performance. An average score was calculated for each task and task scores were averaged across six trials for each participant.

### Statistics

A one-way ANOVA and the post hoc analysis were conducted to compare the average performance score of experts, senior trainees, junior trainees, and novices. A linear regression analysis was conducted to compare trainee average score to that trainee year of training. All data analysis, algorithm training and statistics were carried out using MATLAB (The MathWorks Inc.) release 2020a and IBM SPSS Statistics, Version 27 by codes written by the authors.

### Providing coaching and risk assessment

Three algorithms were developed to provide expert level coaching related to (1) aspirator force utilization, (2) bipolar forceps force utilization, and (3) instrument tip separation distance, outputting these features. While making the predictions for expert-level coaching, the expertise level was inputted as an expert ‘1’ throughout the task. Two other algorithms had output predictions for bleeding and non-tumor tissue injury risks. While making the predictions for risk assessment, the expertise level was inputted aligned with the expertise level of the user (expert: ‘1’, seniors: ‘0.33’, juniors: ‘−0.33’, medical student: ‘−1’). More detailed information about input and output features can be found at the Supplementary Table 2. A future study may address the testing and validation of coaching and risk detection modules of the ICEMS.

### Reporting summary

Further information on research design is available in the Nature Research Reporting Summary linked to this article.

## Supplementary information

Reporting Summary

Supplemental Material

A neurosurgeon’s performance assessed by the ICEMS

A medical student’s performance assessed by the ICEMS

A senior resident’s performance represented with the ICEMS’s assessment, coaching and risk detection modules

A junior resident’s performance represented with the ICEMS’s assessment, coaching and risk detection modules

## Data Availability

The dataset analyzed in this study is available from the corresponding author on a reasonable request. A sample raw simulation data file is available online^61^: 10.6084/m9.figshare.15132507.v1.

## Code availability

The codes written by the authors can be found online: https://github.com/recaiyilmaz/icems.git.
