# Supplementary material for: Continuous monitoring of surgical bimanual expertise using deep neural networks in virtual reality simulation
Source: NPJ Digit Med. 2022 Apr 26;5:54. doi: 10.1038/s41746-022-00596-8 (PMC9042967; doi:10.1038/s41746-022-00596-8)
Supplement: Supplementary file 1 — Reporting Summary [file 41746_2022_596_MOESM1_ESM.pdf]

nature portfolio

Corresponding author(s): Recai Yilmaz

Last updated by author(s): Mar 1, 2022

Reporting Summary

Nature Portfolio wishes to improve the reproducibility of the work that we publish. This form provides structure for consistency and transparency in reporting. For further information on Nature Portfolio policies, see our Editorial Policies and the Editorial Policy Checklist.

Statistics

For all statistical analyses, confirm that the following items are present in the figure legend, table legend, main text, or Methods section.

n/a	Confirmed	
☐	☒	The exact sample size (n) for each experimental group/condition, given as a discrete number and unit of measurement
☐	☒	A statement on whether measurements were taken from distinct samples or whether the same sample was measured repeatedly
☐	☒	The statistical test(s) used AND whether they are one- or two-sided<br>*Only common tests should be described solely by name; describe more complex techniques in the Methods section.*
☒	☐	A description of all covariates tested
☐	☒	A description of any assumptions or corrections, such as tests of normality and adjustment for multiple comparisons
☐	☒	A full description of the statistical parameters including central tendency (e.g. means) or other basic estimates (e.g. regression coefficient) AND variation (e.g. standard deviation) or associated estimates of uncertainty (e.g. confidence intervals)
☐	☒	For null hypothesis testing, the test statistic (e.g. F, t, r) with confidence intervals, effect sizes, degrees of freedom and P value noted<br>*Give P values as exact values whenever suitable.*
☒	☐	For Bayesian analysis, information on the choice of priors and Markov chain Monte Carlo settings
☒	☐	For hierarchical and complex designs, identification of the appropriate level for tests and full reporting of outcomes
☐	☒	Estimates of effect sizes (e.g. Cohen's d, Pearson's r), indicating how they were calculated

Our web collection on statistics for biologists contains articles on many of the points above.

Software and code

Policy information about availability of computer code

Data collection	The NeuroVR surgical simulation platform (CAE Healthcare, Montreal, Quebec, Canada) was utilized to collect data in this study.
Data analysis	All data analysis and algorithm training were carried out using MATLAB (The MathWorks Inc.) release 2020a, by codes written by the corresponding author. Statistics were carried out using IBM SPSS Statistics, Version 27. Codes are publicly available through the link provided in the manuscript.

For manuscripts utilizing custom algorithms or software that are central to the research but not yet described in published literature, software must be made available to editors and reviewers. We strongly encourage code deposition in a community repository (e.g. GitHub). See the Nature Portfolio guidelines for submitting code & software for further information.

Data

Policy information about availability of data

All manuscripts must include a data availability statement. This statement should provide the following information, where applicable:

- Accession codes, unique identifiers, or web links for publicly available datasets
- A description of any restrictions on data availability
- For clinical datasets or third party data, please ensure that the statement adheres to our policy

The dataset analyzed in this study is available from the corresponding author on a reasonable request. A sample raw simulation data file is available online through the link (in reference 61) provided in the manuscript.

Field-specific reporting

Please select the one below that is the best fit for your research. If you are not sure, read the appropriate sections before making your selection.

☐ Life sciences ☒ Behavioural & social sciences ☐ Ecological, evolutionary & environmental sciences

For a reference copy of the document with all sections, see nature.com/documents/nr-reporting-summary-flat.pdf

Behavioural & social sciences study design

All studies must disclose on these points even when the disclosure is negative.

Study description	Neurosurgeons, neurosurgical fellows, neurosurgical residents, and medical students were invited to participate in the trial. Neurosurgery residents indicated the year of training (post-graduate year) they are currently in in their six-year program. All neurosurgical fellows were considered as post-graduate year seven. Performance data was collected through the NeuroVR surgical simulation platform (CAE Healthcare, Montreal, Quebec, Canada). This platform recorded quantitative information during the simulated tasks as described in detail in the manuscript.
Research sample	This study utilized a previously collected data that is currently available at the Neurosurgical Simulation and Artificial Intelligence Learning Centre.
Sampling strategy	This study utilized a previously collected data that belongs to 50 participants. Participants were categorized based on their expertise levels in neurosurgical operative procedures: (1) neurosurgeons, (2) neurosurgical senior residents (post-graduate year 4-6 and neurosurgical fellows), (3) neurosurgical junior residents (post-graduate year 1-3), and (4) medical students.
Data collection	Participants carried out the simulated tumor resections on the NeuroVR platform (CAE Healthcare, Montreal, Quebec, Canada) a total of six times. A researcher accompanied each participant to help them to get familiar with the simulation platform before starting the task. Two simulated tasks were utilized: (1) a practice tumor resection task and (2) complex realistic brain tumor resection task. Participants were given 3 minutes limit for task-1 and 13 minutes limit for task-2. Task-1 was repeated five times and task-2 was done once by the participants.
Timing	The data was collected between March 1, 2015, and May 31, 2016. Data was recorded in a single time point, no-follow up was carried out.
Data exclusions	No data was excluded during the conduct of the study.
Non-participation	No participant declined to participate nor was excluded in this study.
Randomization	Participants were grouped based on whether they are practicing neurosurgeons, or trainees in neurosurgical training or medical students. Trainees in neurosurgical training were separated into two groups: seniors, and juniors. Based on intra-operative exposure, residents who are between post-graduation year (PGY)-4 and PGY-6 and neurosurgical fellows were considered as 'senior trainees'. Residents who are between PGY-1 and PGY-3 were considered as 'junior trainees'.

Reporting for specific materials, systems and methods

We require information from authors about some types of materials, experimental systems and methods used in many studies. Here, indicate whether each material, system or method listed is relevant to your study. If you are not sure if a list item applies to your research, read the appropriate section before selecting a response.

Materials & experimental systems

n/a	Involved in the study	
☒	☐	Antibodies
☒	☐	Eukaryotic cell lines
☒	☐	Palaeontology and archaeology
☒	☐	Animals and other organisms
☐	☒	Human research participants
☒	☐	Clinical data
☒	☐	Dual use research of concern

Methods

n/a	Involved in the study	
☒	☐	ChIP-seq
☒	☐	Flow cytometry
☒	☐	MRI-based neuroimaging

Human research participants

Policy information about studies involving human research participants

Population characteristics	Neurosurgeons, neurosurgical fellows, neurosurgical residents, and medical students from a single institution were invited to participate in the trial.

Recruitment	Neurosurgeons, neurosurgical fellows, neurosurgical residents, and medical students were invited to participate in the trial. The study was conducted to develop an intelligent system to learn participants' existing expert and novice operative skillsets. The medical student cohort included students who may have interest in surgery. The authors believe that the cohort represents the future real-life use of the intelligent system outlined.
Ethics oversight	McGill University Health Centre Research Ethics Board, Neurosciences-Psychiatry

Note that full information on the approval of the study protocol must also be provided in the manuscript.
