# Supplementary material for: Continuous monitoring of surgical bimanual expertise using deep neural networks in virtual reality simulation
Source: NPJ Digit Med. 2022 Apr 26;5:54. doi: 10.1038/s41746-022-00596-8 (PMC9042967; doi:10.1038/s41746-022-00596-8)
Supplement: Supplementary file 2 — Supplemental Material [file 41746_2022_596_MOESM2_ESM.pdf]

Supplementary information

Continuous Monitoring of Surgical Bimanual Expertise Using Deep Neural Networks in Virtual Reality Simulation

Recai Yilmaz[1*], Alexander Winkler-Schwartz[1,2], Nykan Mirchi[1], Aiden Reich[1], Sommer Christie[1], Dan Huy Tran[1], Nicole Ledwos[1], Ali M. Fazlollahi[1], Carlo Santaguida[2], Abdulrahman J. Sabbagh[3,4], Khalid Bajunaid[5], Rolando Del Maestro[1,2]

[1] Neurosurgical Simulation and Artificial Intelligence Learning Centre, Department of Neurology & Neurosurgery, Montreal Neurological Institute, McGill University, 3801 University Street, Room E2.89, H3A 2B4, Montreal, Quebec, Canada.

[2] Department of Neurology and Neurosurgery, Montreal Neurological Institute and hospital, McGill University, Montreal, Quebec, Canada.

[3] Division of Neurosurgery, Department of Surgery, College of Medicine, King Abdulaziz University, Jeddah, Saudi Arabia.

[4] Clinical Skills and Simulation Center, King Abdulaziz University, Jeddah, Saudi Arabia.

[5] Department of Surgery, Faculty of Medicine, University of Jeddah, Jeddah, Saudi Arabia.

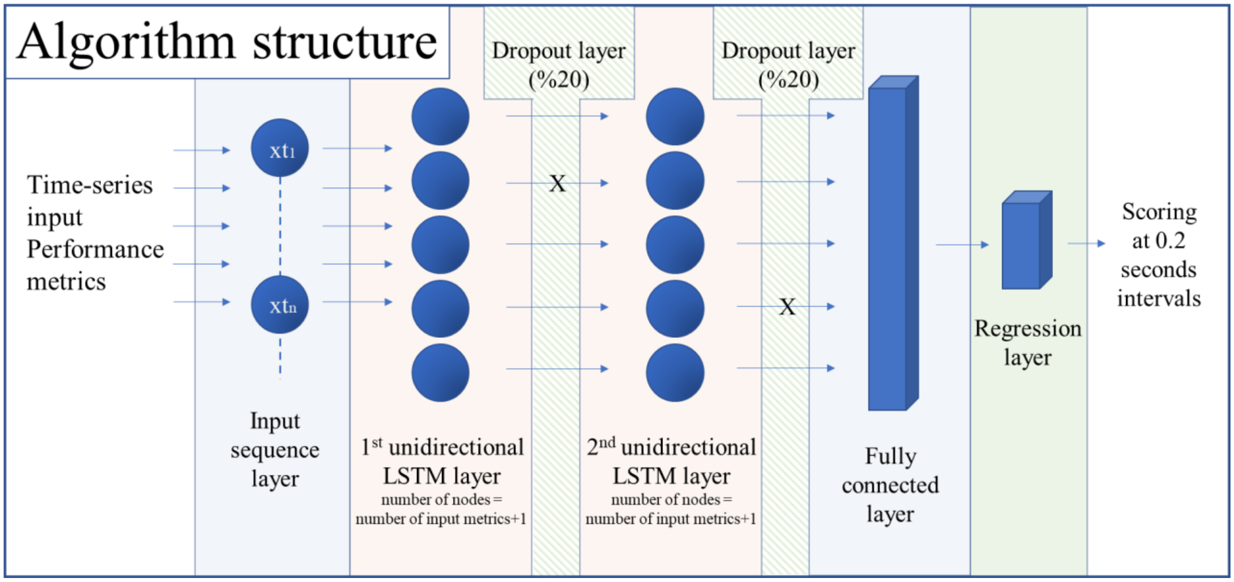

Supplementary Figure 1: Algorithm structure.

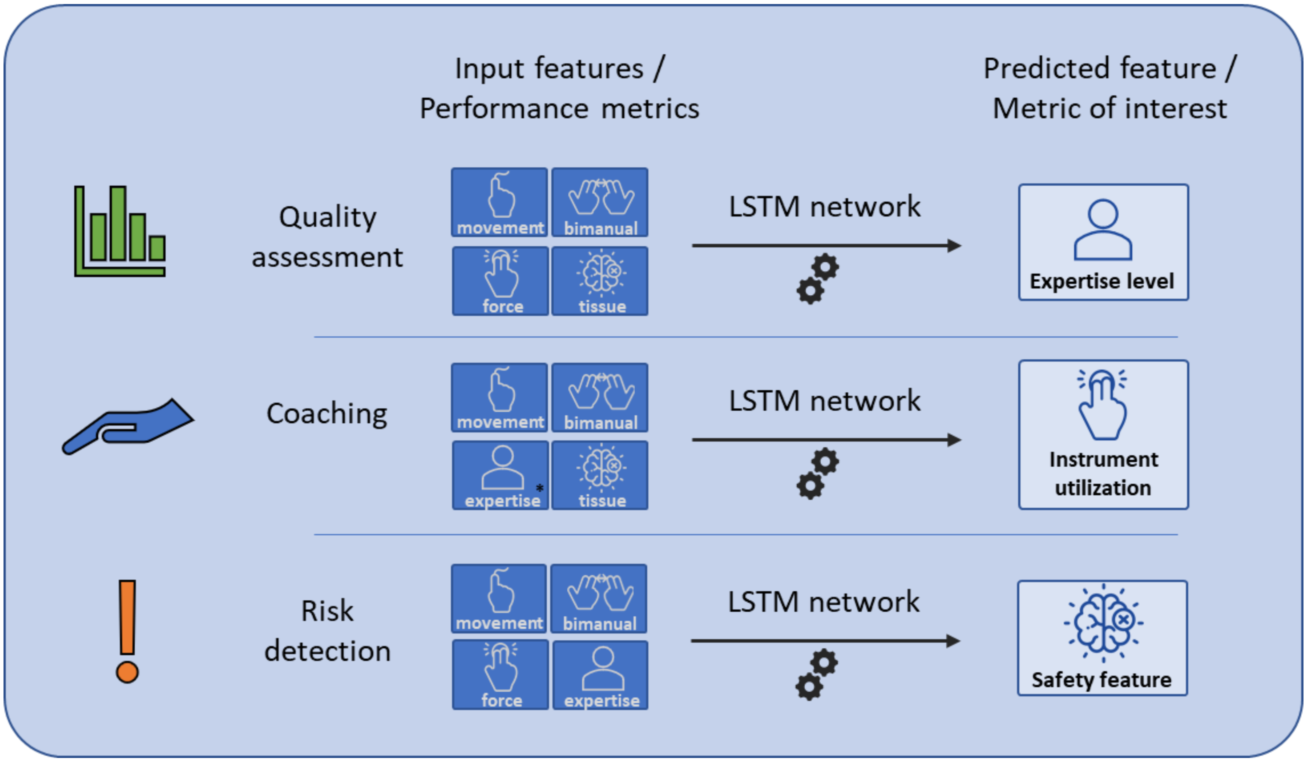

Supplementary Figure 2: Applications of the ICEMS. Our system can be used for three applications. When the expertise level defined as the output feature, a quality assessment of the performance can be made. When a feature relating instrument utilization or operative factor is outputted coaching can be provided (*expertise is inputted as the expert level). When a safety metric defined as the output, a risk detection algorithm can be developed.

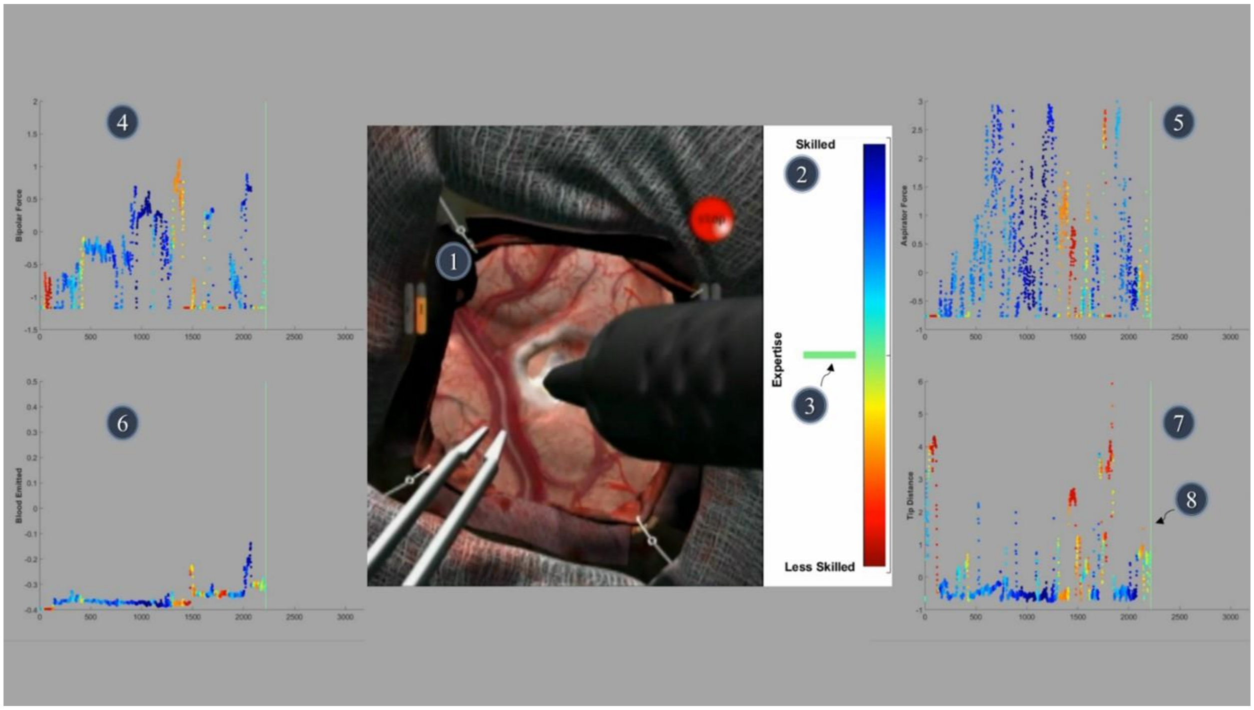

Supplementary Figure 3: Legend of Supplementary Video-1 and Supplementary Video-2. This video represents the expertise assessment made by the ICEMS in relation to 4 of the 16 critical performance metrics inputted to the algorithm. Middle screen **(1)** shows the user view during the virtual reality surgical task. The color bar **(2)** represents the assessment made from skilled -blue- to less skilled -red- levels of expertise, shown by the colored indicator **(3)** at 0.2-second intervals. Four scatter plots, for each critical features including aspirator force **(5)**, bipolar force **(4)**, tip distance **(7)** and blood emitted **(6)**, represent how the expertise assessment relates to these metrics. In these graphs, each dot represents an expertise assessment made by the ICEMS by its color (according to the color bar **(2)**), at each 0.2-second intervals. Colored dots are drawn according to the expertise level determined by the algorithm as the time progress, same color as **(3)** and the colored time indicator **(8)**. x-axis show the number of decisions made. During this >10min task more 3000 assessments were made. y-axis show the z-score values for each performance metric. Higher values indicate higher force applied at **(4)** and **(5)** with bipolar and

aspirator, respectively. High values indicate high bleeding rate at **(6)** and instrument tip separation at **(7)**. The colored time indicator **(8)** proceeds on the y-axis.

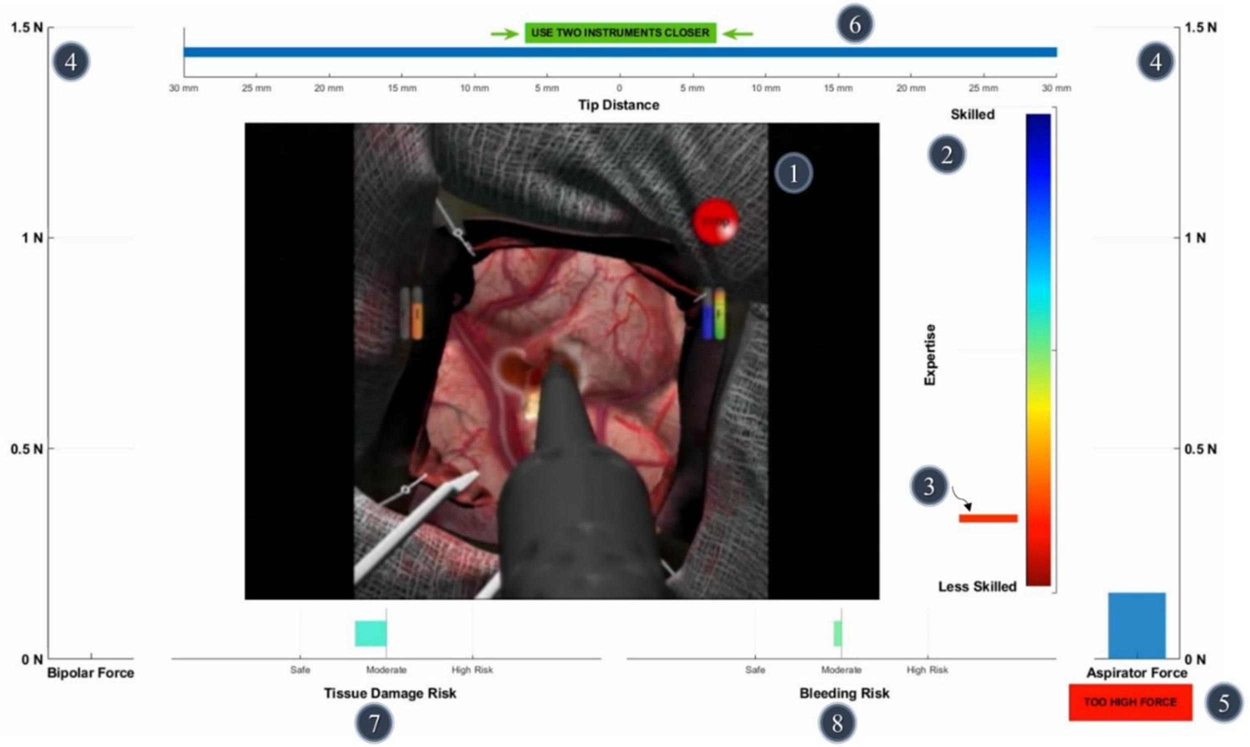

Supplementary Figure 4: Legend of Supplementary Video-3 and Supplementary Video-4.

The ICEMS composed of three modules: assessment, coaching and risk detection. Middle screen **(1)** shows the user view during surgical performance. The color bar **(2)** represents the assessment module where the assessment is made at 0.2-second intervals between skilled -blue- and less skilled -red- levels and shown by the colored indicator **(3)**. In this example coaching is provided for three critical metrics: aspirator force utilized, bipolar forceps force utilized and instrument tip separation distance. The bars **(4)** show the amount of force applied by bipolar **(4-left)** and aspirator **(4-right)**. Two background algorithms calculate the expected force applied for expert level instrument utilization. If the expected value is one standard deviation below the actual value a warning **(5)** ‘too high force’ is given. If the expected value is one standard deviation above the actual value a warning ‘use bipolar/aspirator more efficiently’ is given. The top bar **(6)** shows the distance between the tip of the two instruments. A background algorithm calculates

the expected tip separation distance for expert level instrument utilization. If the expected level is one standard deviation below the actual value, a warning ‘use two instruments together’ is shown. The risks related to two critical features were detected: tissue (healthy brain) damage risk **(7)**, and bleeding risk **(8)**. The moderate risk level equals the average risk achieved by all individuals within our dataset, where z-score=0. Higher values indicated behaviour with high risk and lower values indicated safe behaviour.

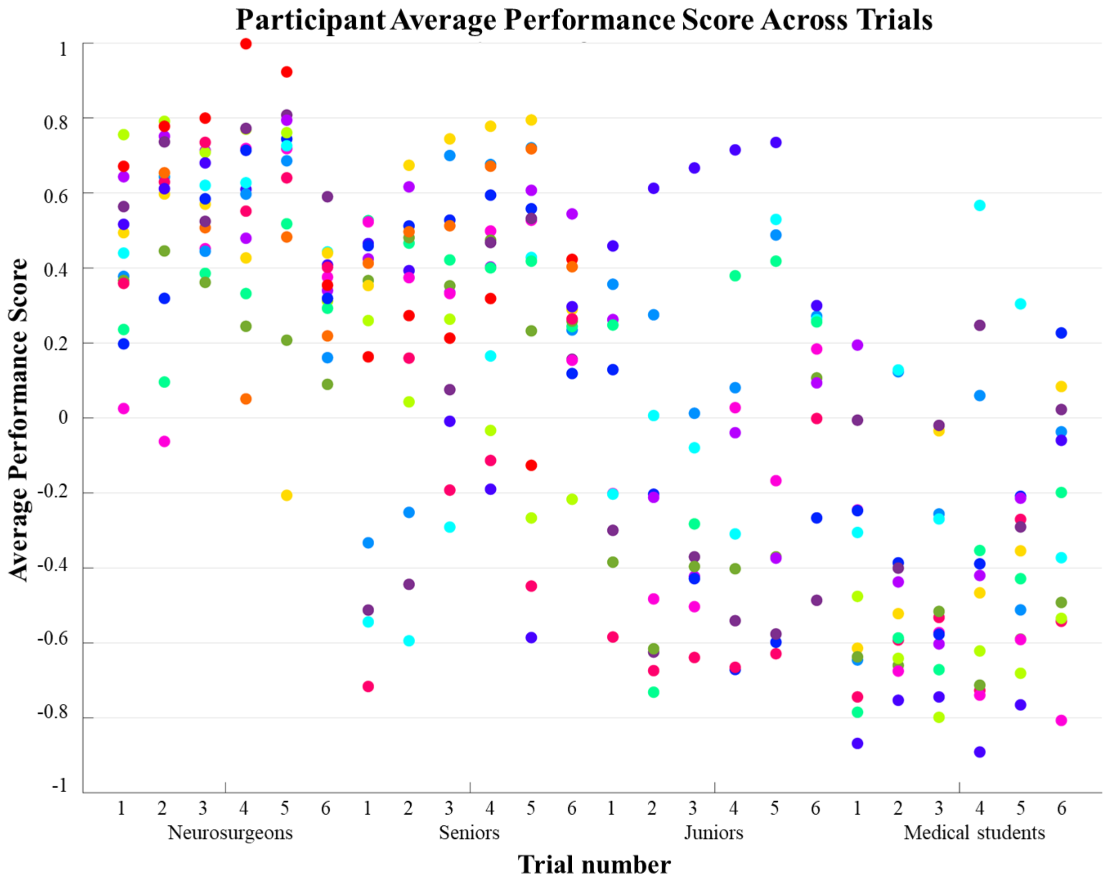

Supplementary Figure 5: Participant Average Performance Score Across Trials. X-axis represents the trial numbers from first to sixth repeat for each expertise group. Trial number 1 to 5 belongs to the practice trial while trial number 6 indicates the realistic scenario. Y-axis represents the average performance score. Participant scores at each task is indicated with a colored dot. Same color was utilized within the same expertise group for each participant. Data that belongs to a neurosurgeon for the fifth repeat was not available.

	TRAINING RMSE	VALIDATION RMSE	TESTING RMSE
**Expertise**	0.7065	0.7097	0.7525
**Force Aspirator**	0.8169	1.0648	0.8994
**Force Bipolar**	0.6859	0.7939	0.6647
**Tip Distance**	0.6611	0.7166	0.6786
**Tissue Damage**	0.8791	0.8377	0.7998
**Bleeding**	0.6910	0.6200	0.6099

RMSE: root-mean-squared-error

Supplementary Table 1: Root-mean-squared-error (RMSE) values obtained. A total of six algorithms were trained for assessment, coaching and risk detection. The training accuracy was monitored by root-mean-squared error (RMSE) values. During algorithm training, overfitting happens when the model fits a dataset too closely preventing accurate prediction on a new dataset (low generalizability). To avoid this problem the separate validation dataset was used to monitor the training progress. A training was acceptable when the RMSEs for training and validation datasets decreased in tandem and stay aligned by the end of the training. After the training was complete, the separate testing dataset was used to check the final state of the training. Having no gold standard, close values for training, validation and testing were targeted to help reject overfitting.

Assessment made	Input features	Output feature	Explanation
Performance assessment	**1**, **2**, **3**, **4**, **5**, **6**, **7**, **8**, **9**, **10**, **11**, **12**, **13**, **14**, **15**, **16**	**0**- Expertise	This algorithm makes a global expertise assessment given the 16 input features.
Aspirator utilization	**0***, **2**, **3**, **5**, **6**, **7**, **8**, **9**, **10**, **11**, **12**, **13**, **14**, **15**, **16**<br><br>*inputted as always '1'.<br>Excluded **4** because the output feature is a parent feature.	**1**- Force Utilized by Aspirator	This algorithm predicts the amount of force utilization expected for expert level ('1'), for specific action being performed. Predicted values can be used to coach a trainee. The output metric represents a safety and efficiency feature. A trainee is expected use aspirator efficiently while avoiding high forces.
Bipolar forceps utilization	**0***, **1**, **3**, **4**, **6**, **7**, **8**, **9**, **10**, **11**, **12**, **13**, **14**, **15**, **16**<br><br>*inputted as always '1'.<br>Excluded **5** because the output feature is a parent feature.	**2**- Force Utilized by Bipolar Forceps	This algorithm predicts the amount of force utilization expected for expert level ('1'), for specific action being performed. Predicted values can be used to coach a trainee. The output metric represents a safety and efficiency feature. A trainee is expected use bipolar forceps efficiently while avoiding high forces.
Bimanual instrument utilization	**0***, **1**, **2**, **4**, **5**, **6**, **7**, **8**, **9**, **11**, **12**, **13**, **14**, **15**, **16**<br><br>*inputted as always '1'.<br>Excluded **10** because the output feature is a parent feature.	**3**- Instrument Tip Separation Distance	This algorithm predicts how close the two instruments should be during the action being performed for expert level ('1'). Predicted values can be used to coach a trainee. The output metric represents the bimanual cognitive. A trainee is expected use the tips of the two instruments closely together.
Bleeding risk detection	**0***, **1**, **2**, **3**, **4**, **5**, **6**, **7**, **8**, **9**, **10**, **11**, **12**<br><br>*inputted aligned with the expertise level of the user (expert: '1', seniors: '0.33', juniors: '-0.33', medical student: '-1').<br>Excluded **14**, **15**, **16** because the output feature is a closely related feature.	**13**- Bleeding Speed	This algorithm predicts bleeding rate during the action being performed. The output metric represents a safety feature.
Tissue damage risk detection	**0***, **1**, **2**, **3**, **4**, **5**, **6**, **7**, **8**, **9**, **10**, **11**, **13**, **14**, **15**, **16**<br><br>*inputted aligned with the expertise level of the user (expert: '1', seniors: '0.33', juniors: '-0.33', medical student: '-1').<br>No feature exclusion was made.	**12**- Healthy Tissue Removed	This algorithm predicts damage to the healthy surrounding tissue during the action being performed. The output metric represents a safety feature.
**Number coded features:** **0**- Expertise, **1**- Force Utilized by Aspirator, **2**- Force Utilized by Bipolar Forceps, **3**- Instrument Tip Separation Distance, **4**- Force Change Aspirator, **5**- Force Change Bipolar Forceps, **6**- Aspirator Velocity, **7**- Bipolar Forceps Velocity, **8**- Aspirator Acceleration, **9**- Bipolar Forceps Acceleration, **10**- Instrument Tip Separation Distance Change, **11**- Tumor Volume Removed, **12**- Healthy Tissue Removed, **13**- Bleeding Speed, **14**- Blood Pooling, **15**- Total Blood Loss, **16**- Blood Pooling Change			

Supplementary Table 2: Input and output features (metric of interest) for each trained algorithm. Colors indicate the three categories of application: (1- green) expertise assessment, (2- blue) coaching, and (3- red) risk assessment.

	Trainee ID	Post Graduate Year of Training	Number of times assisted in subpial resection	Number of times carried out partial subpial resection	Number of times carried out complete subpial resection	Average Score
Neurosurgical Fellows	1	7	110	85	32	0.488
	2	7	4	4	4	0.461
	3	7	31	22	34	0.361
	4	7	36	4	6	0.046
Neurosurgical Senior Residents	5	6	0	0	2	-0.175
	6	6	24	16	12	0.401
	7	6	0	20	45	0.062
	8	5	24	20	7	-0.096
	9	5	133	130	5	0.605
	10	4	18	3	0	0.291
	11	4	2	1	0	0.413
	12	4	3	1	0	0.008
	13	4	57	30	10	0.536
	14	4	2	2	0	0.211
Neurosurgical Junior Residents	15	3	3	1	0	-0.191
	16	3	8	35	7	-0.115
	17	3	15	12	3	0.581
	18	3	0	0	0	0.247
	19	2	3	0	0	-0.532
	20	2	1	0	0	0.048
	21	1	0	0	0	-0.340
	22	1	0	0	0	0.034
	23	1	1	2	0	-0.344
	24	1	24	0	0	-0.483

Supplementary Table 3: Trainee self-reported subpial resection operative experience and trainee average ICEMS scores. Trainees reported the number of subpial procedures they involved in, including epilepsy cases, and frontal, temporal, and occipital brain tumor surgical procedures. Right-most column shows the trainee average expertise score rated across six simulation trials by the ICEMS. Neurosurgical fellows were considered in 7th year in training.
